# Triclinic polymorph of bis­(triphenyl­sil­yl) oxide toluene disolvate

**DOI:** 10.1107/S160053681200356X

**Published:** 2012-02-04

**Authors:** Andrew P. Purdy, Emily Smoot, Ray J. Butcher, Andrew Kerr

**Affiliations:** a Naval Research Laboratory, Chemistry Division, Code 6100, 4555 Overlook Avenue SW, Washington, DC 20375, USA; b Department of Chemistry, Howard University, 525 College Street NW, Washington, DC 20059, USA; c George Washington University, Chemistry Department, Washington, DC, USA

## Abstract

A new polymorph of the title compound, C_36_H_30_OSi_2_·2C_7_H_8_, is reported, which is triclinic (*P*-1) instead of possessing the previously reported rhombohedral symmetry [Hönle *et al.* (1990). *Acta Cryst.* C**46**, 1982–1984]. Each of the –SiPh_3_ units are related by the inversion center. The Si—O—Si moiety is linear with the O atom sitting on an inversion center, and the O—Si—(toluene ring centroid) angle is 3.69 (15)°. Each toluene mol­ecule is 5.622 (2) Å from the Si atom and has its closest contacts with the phenyl rings outside of the van der Waals radii.

## Related literature

For the rhombohedral polymorph of the title compound and its benzene analog, see: Hönle *et al.* (1990 ▶). For the structures of related compounds, see: Glidewell & Liles (1978 ▶); Morosin & Harrah (1981 ▶); Suwińska *et al.* (1986 ▶). For the determination by IR spectroscopy of silylcarbonate in the reaction product, see: Yildirimyan & Gattow (1984 ▶).
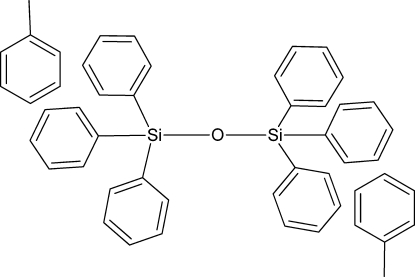


## Experimental

### 

#### Crystal data

C_36_H_30_OSi_2_·2C_7_H_8_
*M* *_r_* = 719.05
Triclinic, 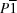

*a* = 10.756 (3) Å
*b* = 11.062 (3) Å
*c* = 11.156 (3) Å
α = 62.638 (4)°
β = 61.356 (4)°
γ = 61.795 (4)°
*V* = 978.8 (4) Å^3^
*Z* = 1
Mo *K*α radiation
μ = 0.13 mm^−1^
*T* = 123 K
0.33 × 0.25 × 0.12 mm

#### Data collection

Bruker APEXII CCD diffractometer
Absorption correction: multi-scan (*SADABS*; Bruker, 2005 ▶) *T*_min_ = 0.605, *T*_max_ = 0.746
16663 measured reflections
4819 independent reflections
3344 reflections with *I* > 2σ(*I*)
*R*_int_ = 0.057

#### Refinement

*R*[*F* ^2^ > 2σ(*F* ^2^)] = 0.088
*wR*(*F* ^2^) = 0.259
*S* = 1.06
4819 reflections
242 parameters
H-atom parameters constrained
Δρ_max_ = 1.38 e Å^−3^ (near atom Si1)
Δρ_min_ = −0.52 e Å^−3^

### 

Data collection: *APEX2* (Bruker, 2005 ▶); cell refinement: *SAINT* (Bruker, 2005 ▶); data reduction: *SAINT*; program(s) used to solve structure: *SHELXS97* (Sheldrick, 2008 ▶); program(s) used to refine structure: *SHELXL97* (Sheldrick, 2008 ▶); molecular graphics: *SHELXTL* (Sheldrick, 2008 ▶); software used to prepare material for publication: *SHELXTL*.

## Supplementary Material

Crystal structure: contains datablock(s) I, global. DOI: 10.1107/S160053681200356X/rz2678sup1.cif

Structure factors: contains datablock(s) I. DOI: 10.1107/S160053681200356X/rz2678Isup2.hkl

Supplementary material file. DOI: 10.1107/S160053681200356X/rz2678Isup3.cml

Additional supplementary materials:  crystallographic information; 3D view; checkCIF report

## Figures and Tables

**Table:** 

Si1—O1	1.6251 (9)
Si1—C7	1.863 (4)
Si1—C1	1.865 (4)
Si1—C13	1.867 (4)

**Table:** 

O1—Si1—C7	107.86 (11)
O1—Si1—C1	108.74 (12)
C7—Si1—C1	110.50 (16)
O1—Si1—C13	108.28 (11)
C7—Si1—C13	111.99 (16)
C1—Si1—C13	109.38 (16)
Si1—O1—Si1^i^	180

Symmetry code: (i) 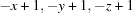.

**Fig. 1.**
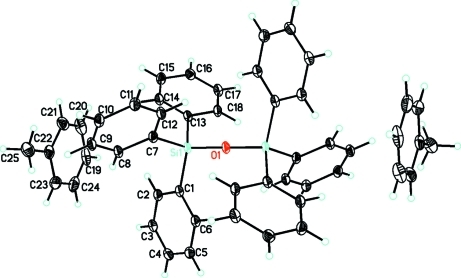
Structural diagram of the title compound. Atomic displacement parameters are at the 30% probability level. Unlabelled atoms are related to the labelled atoms by the symmetry operation 1-x, 1-y, 1-z.

## supplementary crystallographic information

### Comment

A rhombohedral polymorph of the title compound and its benzene analog have been reported by Hönle *et al.* (1990). The triclinic unsolvated molecule was reported by Glidewell & Liles (1978) and repeated by Morosin & Harrah (1981), who also determined the entire series of Ge and Sn analogs. A benzene and a piperidine adduct of (Ph_3_Si)_2_O were also determined by Suwińska *et al.* (1986).

The core geometry (Table 1) of the title compound is almost identical to the previously reported rhombohedral polymorph. The main difference is the rhombohedral form was collected at room temperature, and has rotational disorder in the toluene. Our structure was collected at -150°C, and the toluene is not disordered. This difference could account for the lower symmetry of our structure. In our structure, the centroid of the toluene is slightly offset (3.69 (15)° *versus* 0°) from the linear Si—O—Si axis, and slightly closer (5.622 (5) *versus* 5.672 (2) Å) than it is in the rhombohedral form.

### Experimental

All chemicals were handled inside a dry box under Ar or N_2_. A Ph_3_SiOK solution was prepared by dissolving KH (0.28 g) with Ph_3_SiOH (2.0 g) in 30 g of dry ether, and filtering to remove the small amount of undissolved solids. The solution was put in a 1" diameter test tube which was inserted into a Newport Scientific 2" OD pressure vessel and pressurized with CO_2_ at tank pressure. After 1 day, the pressure was released and the resulting voluminous white solid (presumably Ph_3_SiOCO_2_K) was isolated by filtration and washing with ether, and pumped dry under vacuum. An infrared spectrum of the white solid shows a broad peak at 1660 cm^-1^ and is consistent (Yildirimyan & Gattow, 1984) with the presence of silylcarbonate. Attempts were made to crystallize the silylcarbonate from numerous solvents. In one attempt, a portion of the white solid was mixed in a vial with toluene and 18-crown-6. Over several weeks small crystals formed on the walls of the vial. One crystal was mounted in Cargill #2 oil and frozen in the diffractometer.

### Refinement

H atoms were placed in geometrically idealized positions and constrained to ride on their parent atoms with a C—H distance of 0.95 and 0.98 Å *U*_iso_(H) = 1.2*U*_eq_(C) [1.5*U*_eq_(C) for CH_3_]. During data collection it was found that the previous crystal (a completely unrelated material) was still present on the MiTiGen mount. However since its cell constants were different from those of the current compound, there was very little overlap of the reflections. The unit cell for the title compound was determined using the twinning routine cell_now and integration proceeded smoothly.

### Crystal data

**Table:**

C_36_H_30_OSi_2_·2C_7_H_8_	*Z* = 1
*M**_r_* = 719.05	*F*(000) = 382
Triclinic, *P*1	*D*_x_ = 1.220 Mg m^−^^3^
*a* = 10.756 (3) Å	Mo *K*α radiation, λ = 0.71073 Å
*b* = 11.062 (3) Å	Cell parameters from 8183 reflections
*c* = 11.156 (3) Å	θ = 2.3–28.3°
α = 62.638 (4)°	µ = 0.13 mm^−^^1^
β = 61.356 (4)°	*T* = 123 K
γ = 61.795 (4)°	Prism, colorless
*V* = 978.8 (4) Å^3^	0.33 × 0.25 × 0.12 mm

### Data collection

**Table:**

Bruker APEXII CCD diffractometer	4819 independent reflections
Radiation source: fine-focus sealed tube	3344 reflections with *I* > 2σ(*I*)
Graphite monochromator	*R*_int_ = 0.057
φ and ω scans	θ_max_ = 28.5°, θ_min_ = 2.2°
Absorption correction: multi-scan (*SADABS*; Bruker, 2005)	*h* = −14→14
*T*_min_ = 0.605, *T*_max_ = 0.746	*k* = −14→14
16663 measured reflections	*l* = −14→14

### Refinement

**Table:**

Refinement on *F*^2^	Primary atom site location: structure-invariant direct methods
Least-squares matrix: full	Secondary atom site location: difference Fourier map
*R*[*F*^2^ > 2σ(*F*^2^)] = 0.088	Hydrogen site location: inferred from neighbouring sites
*wR*(*F*^2^) = 0.259	H-atom parameters constrained
*S* = 1.06	*w* = 1/[σ^2^(*F*_o_^2^) + (0.1084*P*)^2^ + 2.5305*P*] where *P* = (*F*_o_^2^ + 2*F*_c_^2^)/3
4819 reflections	(Δ/σ)_max_ < 0.001
242 parameters	Δρ_max_ = 1.38 e Å^−^^3^
0 restraints	Δρ_min_ = −0.52 e Å^−^^3^

### Special details

**Table:**

Geometry. All e.s.d.'s (except the e.s.d. in the dihedral angle between two l.s. planes) are estimated using the full covariance matrix. The cell e.s.d.'s are taken into account individually in the estimation of e.s.d.'s in distances, angles and torsion angles; correlations between e.s.d.'s in cell parameters are only used when they are defined by crystal symmetry. An approximate (isotropic) treatment of cell e.s.d.'s is used for estimating e.s.d.'s involving l.s. planes.
Refinement. Refinement of *F*^2^ against ALL reflections. The weighted *R*-factor *wR* and goodness of fit *S* are based on *F*^2^, conventional *R*-factors *R* are based on *F*, with *F* set to zero for negative *F*^2^. The threshold expression of *F*^2^ > σ(*F*^2^) is used only for calculating *R*-factors(gt) *etc*. and is not relevant to the choice of reflections for refinement. *R*-factors based on *F*^2^ are statistically about twice as large as those based on *F*, and *R*- factors based on ALL data will be even larger.

### Fractional atomic coordinates and isotropic or equivalent isotropic displacement parameters (Å^2^)

**Table:**

	*x*	*y*	*z*	*U*_iso_*/*U*_eq_	
Si1	0.43554 (11)	0.43924 (10)	0.44133 (11)	0.0247 (3)	
O1	0.5000	0.5000	0.5000	0.0275 (8)	
C1	0.5919 (4)	0.3631 (4)	0.2956 (4)	0.0269 (7)	
C2	0.5652 (4)	0.3559 (4)	0.1875 (4)	0.0294 (8)	
H2	0.4654	0.3901	0.1888	0.035*	
C3	0.6820 (5)	0.2998 (4)	0.0797 (4)	0.0316 (8)	
H3	0.6619	0.2960	0.0076	0.038*	
C4	0.8287 (5)	0.2491 (4)	0.0760 (4)	0.0323 (8)	
H4	0.9085	0.2110	0.0014	0.039*	
C5	0.8585 (5)	0.2541 (4)	0.1816 (4)	0.0332 (8)	
H5	0.9587	0.2187	0.1799	0.040*	
C6	0.7411 (4)	0.3110 (4)	0.2898 (4)	0.0294 (8)	
H6	0.7623	0.3148	0.3613	0.035*	
C7	0.3593 (4)	0.2968 (4)	0.5940 (4)	0.0262 (7)	
C8	0.3663 (4)	0.1750 (4)	0.5763 (4)	0.0285 (7)	
H8	0.4073	0.1664	0.4827	0.034*	
C9	0.3140 (4)	0.0672 (4)	0.6935 (4)	0.0325 (8)	
H9	0.3201	−0.0140	0.6792	0.039*	
C10	0.2535 (4)	0.0779 (4)	0.8303 (4)	0.0309 (8)	
H10	0.2192	0.0036	0.9101	0.037*	
C11	0.2428 (4)	0.1985 (4)	0.8507 (4)	0.0319 (8)	
H11	0.1996	0.2074	0.9443	0.038*	
C12	0.2958 (4)	0.3052 (4)	0.7335 (4)	0.0301 (8)	
H12	0.2887	0.3864	0.7487	0.036*	
C13	0.2885 (4)	0.5928 (4)	0.3676 (4)	0.0266 (7)	
C14	0.1678 (4)	0.5759 (4)	0.3677 (4)	0.0302 (8)	
H14	0.1561	0.4836	0.4105	0.036*	
C15	0.0640 (4)	0.6927 (4)	0.3057 (4)	0.0325 (8)	
H15	−0.0171	0.6793	0.3060	0.039*	
C16	0.0790 (4)	0.8284 (4)	0.2436 (4)	0.0316 (8)	
H16	0.0074	0.9081	0.2028	0.038*	
C17	0.1988 (5)	0.8474 (4)	0.2413 (4)	0.0330 (8)	
H17	0.2103	0.9399	0.1974	0.040*	
C18	0.3021 (4)	0.7311 (4)	0.3031 (4)	0.0296 (8)	
H18	0.3833	0.7453	0.3017	0.035*	
C19	0.2596 (7)	0.3594 (5)	0.0656 (6)	0.0559 (14)	
H19	0.2725	0.4353	−0.0217	0.067*	
C20	0.1304 (7)	0.3834 (6)	0.1816 (6)	0.0571 (14)	
H20	0.0550	0.4750	0.1735	0.068*	
C21	0.1120 (6)	0.2751 (5)	0.3072 (5)	0.0450 (11)	
H21	0.0239	0.2922	0.3869	0.054*	
C22	0.2219 (5)	0.1381 (5)	0.3207 (5)	0.0378 (9)	
C23	0.3501 (5)	0.1166 (5)	0.2024 (5)	0.0437 (10)	
H23	0.4255	0.0249	0.2091	0.052*	
C24	0.3695 (6)	0.2270 (6)	0.0751 (6)	0.0528 (13)	
H24	0.4578	0.2116	−0.0050	0.063*	
C25	0.2010 (6)	0.0204 (5)	0.4542 (5)	0.0469 (11)	
H25A	0.1330	0.0600	0.5345	0.070*	
H25B	0.2979	−0.0403	0.4682	0.070*	
H25C	0.1582	−0.0375	0.4492	0.070*	

### Atomic displacement parameters (Å^2^)

**Table:**

	*U*^11^	*U*^22^	*U*^33^	*U*^12^	*U*^13^	*U*^23^
Si1	0.0311 (5)	0.0208 (4)	0.0317 (5)	−0.0106 (4)	−0.0141 (4)	−0.0089 (4)
O1	0.0334 (19)	0.0253 (17)	0.036 (2)	−0.0116 (14)	−0.0158 (17)	−0.0114 (14)
C1	0.0346 (18)	0.0219 (15)	0.0316 (19)	−0.0113 (13)	−0.0137 (16)	−0.0087 (13)
C2	0.0345 (19)	0.0269 (17)	0.035 (2)	−0.0122 (14)	−0.0147 (17)	−0.0102 (14)
C3	0.042 (2)	0.0286 (17)	0.035 (2)	−0.0145 (15)	−0.0152 (18)	−0.0118 (15)
C4	0.039 (2)	0.0276 (17)	0.033 (2)	−0.0119 (15)	−0.0097 (17)	−0.0131 (15)
C5	0.0340 (19)	0.0305 (18)	0.041 (2)	−0.0106 (15)	−0.0154 (18)	−0.0121 (16)
C6	0.0358 (19)	0.0255 (16)	0.035 (2)	−0.0111 (14)	−0.0159 (17)	−0.0100 (14)
C7	0.0297 (17)	0.0244 (16)	0.0332 (19)	−0.0098 (13)	−0.0155 (16)	−0.0089 (13)
C8	0.0347 (19)	0.0236 (16)	0.035 (2)	−0.0106 (14)	−0.0147 (17)	−0.0106 (14)
C9	0.037 (2)	0.0214 (16)	0.045 (2)	−0.0112 (14)	−0.0167 (19)	−0.0103 (15)
C10	0.0355 (19)	0.0235 (16)	0.038 (2)	−0.0135 (14)	−0.0174 (18)	−0.0033 (14)
C11	0.037 (2)	0.0336 (19)	0.031 (2)	−0.0172 (16)	−0.0101 (17)	−0.0103 (15)
C12	0.0354 (19)	0.0262 (17)	0.037 (2)	−0.0128 (14)	−0.0130 (17)	−0.0124 (14)
C13	0.0323 (18)	0.0230 (16)	0.0314 (19)	−0.0098 (13)	−0.0130 (16)	−0.0102 (13)
C14	0.0360 (19)	0.0242 (16)	0.038 (2)	−0.0115 (14)	−0.0164 (17)	−0.0091 (14)
C15	0.036 (2)	0.0335 (19)	0.039 (2)	−0.0138 (16)	−0.0182 (18)	−0.0100 (16)
C16	0.0348 (19)	0.0298 (18)	0.035 (2)	−0.0072 (15)	−0.0169 (17)	−0.0115 (15)
C17	0.043 (2)	0.0244 (17)	0.039 (2)	−0.0115 (15)	−0.0210 (19)	−0.0079 (15)
C18	0.0347 (19)	0.0245 (16)	0.039 (2)	−0.0123 (14)	−0.0170 (17)	−0.0089 (14)
C19	0.099 (4)	0.046 (3)	0.052 (3)	−0.044 (3)	−0.036 (3)	−0.005 (2)
C20	0.086 (4)	0.043 (3)	0.061 (3)	−0.023 (3)	−0.037 (3)	−0.015 (2)
C21	0.050 (3)	0.045 (2)	0.055 (3)	−0.011 (2)	−0.022 (2)	−0.026 (2)
C22	0.049 (2)	0.043 (2)	0.039 (2)	−0.0232 (19)	−0.016 (2)	−0.0157 (17)
C23	0.040 (2)	0.052 (3)	0.054 (3)	−0.0162 (19)	−0.018 (2)	−0.024 (2)
C24	0.060 (3)	0.072 (3)	0.050 (3)	−0.044 (3)	−0.009 (3)	−0.022 (2)
C25	0.059 (3)	0.044 (2)	0.044 (3)	−0.017 (2)	−0.020 (2)	−0.016 (2)

### Geometric parameters (Å, º)

**Table:**

Si1—O1	1.6251 (9)	C13—C14	1.396 (5)
Si1—C7	1.863 (4)	C13—C18	1.407 (5)
Si1—C1	1.865 (4)	C14—C15	1.396 (5)
Si1—C13	1.867 (4)	C14—H14	0.9500
O1—Si1^i^	1.6251 (9)	C15—C16	1.387 (5)
C1—C6	1.404 (5)	C15—H15	0.9500
C1—C2	1.409 (5)	C16—C17	1.387 (5)
C2—C3	1.382 (5)	C16—H16	0.9500
C2—H2	0.9500	C17—C18	1.390 (5)
C3—C4	1.388 (6)	C17—H17	0.9500
C3—H3	0.9500	C18—H18	0.9500
C4—C5	1.391 (5)	C19—C24	1.376 (8)
C4—H4	0.9500	C19—C20	1.385 (8)
C5—C6	1.391 (5)	C19—H19	0.9500
C5—H5	0.9500	C20—C21	1.361 (7)
C6—H6	0.9500	C20—H20	0.9500
C7—C12	1.397 (5)	C21—C22	1.409 (6)
C7—C8	1.413 (4)	C21—H21	0.9500
C8—C9	1.394 (5)	C22—C23	1.391 (6)
C8—H8	0.9500	C22—C25	1.462 (6)
C9—C10	1.381 (6)	C23—C24	1.384 (7)
C9—H9	0.9500	C23—H23	0.9500
C10—C11	1.398 (5)	C24—H24	0.9500
C10—H10	0.9500	C25—H25A	0.9800
C11—C12	1.390 (5)	C25—H25B	0.9800
C11—H11	0.9500	C25—H25C	0.9800
C12—H12	0.9500		
			
O1—Si1—C7	107.86 (11)	C14—C13—C18	117.9 (3)
O1—Si1—C1	108.74 (12)	C14—C13—Si1	122.9 (3)
C7—Si1—C1	110.50 (16)	C18—C13—Si1	119.2 (3)
O1—Si1—C13	108.28 (11)	C15—C14—C13	121.0 (3)
C7—Si1—C13	111.99 (16)	C15—C14—H14	119.5
C1—Si1—C13	109.38 (16)	C13—C14—H14	119.5
Si1—O1—Si1^i^	180	C16—C15—C14	120.2 (3)
C6—C1—C2	117.6 (3)	C16—C15—H15	119.9
C6—C1—Si1	120.9 (3)	C14—C15—H15	119.9
C2—C1—Si1	121.5 (3)	C15—C16—C17	119.8 (3)
C3—C2—C1	121.0 (3)	C15—C16—H16	120.1
C3—C2—H2	119.5	C17—C16—H16	120.1
C1—C2—H2	119.5	C16—C17—C18	120.0 (3)
C2—C3—C4	120.5 (3)	C16—C17—H17	120.0
C2—C3—H3	119.8	C18—C17—H17	120.0
C4—C3—H3	119.8	C17—C18—C13	121.2 (3)
C3—C4—C5	119.9 (4)	C17—C18—H18	119.4
C3—C4—H4	120.1	C13—C18—H18	119.4
C5—C4—H4	120.1	C24—C19—C20	121.0 (5)
C4—C5—C6	119.7 (4)	C24—C19—H19	119.5
C4—C5—H5	120.1	C20—C19—H19	119.5
C6—C5—H5	120.1	C21—C20—C19	119.7 (5)
C5—C6—C1	121.3 (3)	C21—C20—H20	120.2
C5—C6—H6	119.3	C19—C20—H20	120.2
C1—C6—H6	119.3	C20—C21—C22	121.0 (5)
C12—C7—C8	117.0 (3)	C20—C21—H21	119.5
C12—C7—Si1	120.3 (2)	C22—C21—H21	119.5
C8—C7—Si1	122.6 (3)	C23—C22—C21	118.2 (4)
C9—C8—C7	121.2 (3)	C23—C22—C25	120.6 (4)
C9—C8—H8	119.4	C21—C22—C25	121.2 (4)
C7—C8—H8	119.4	C24—C23—C22	120.9 (5)
C10—C9—C8	120.3 (3)	C24—C23—H23	119.6
C10—C9—H9	119.8	C22—C23—H23	119.6
C8—C9—H9	119.8	C19—C24—C23	119.3 (5)
C9—C10—C11	119.7 (3)	C19—C24—H24	120.3
C9—C10—H10	120.2	C23—C24—H24	120.3
C11—C10—H10	120.2	C22—C25—H25A	109.5
C12—C11—C10	119.7 (4)	C22—C25—H25B	109.5
C12—C11—H11	120.1	H25A—C25—H25B	109.5
C10—C11—H11	120.1	C22—C25—H25C	109.5
C11—C12—C7	122.0 (3)	H25A—C25—H25C	109.5
C11—C12—H12	119.0	H25B—C25—H25C	109.5
C7—C12—H12	119.0		
			
O1—Si1—C1—C6	25.8 (3)	C10—C11—C12—C7	0.5 (6)
C7—Si1—C1—C6	−92.4 (3)	C8—C7—C12—C11	0.6 (5)
C13—Si1—C1—C6	143.9 (3)	Si1—C7—C12—C11	−177.6 (3)
O1—Si1—C1—C2	−154.1 (3)	O1—Si1—C13—C14	−150.6 (3)
C7—Si1—C1—C2	87.7 (3)	C7—Si1—C13—C14	−31.8 (4)
C13—Si1—C1—C2	−36.0 (3)	C1—Si1—C13—C14	91.1 (3)
C6—C1—C2—C3	−0.2 (5)	O1—Si1—C13—C18	32.4 (3)
Si1—C1—C2—C3	179.7 (3)	C7—Si1—C13—C18	151.2 (3)
C1—C2—C3—C4	0.1 (5)	C1—Si1—C13—C18	−86.0 (3)
C2—C3—C4—C5	0.2 (6)	C18—C13—C14—C15	−0.1 (6)
C3—C4—C5—C6	−0.5 (6)	Si1—C13—C14—C15	−177.1 (3)
C4—C5—C6—C1	0.5 (6)	C13—C14—C15—C16	−0.4 (6)
C2—C1—C6—C5	−0.1 (5)	C14—C15—C16—C17	1.0 (6)
Si1—C1—C6—C5	180.0 (3)	C15—C16—C17—C18	−1.1 (6)
O1—Si1—C7—C12	31.8 (3)	C16—C17—C18—C13	0.6 (6)
C1—Si1—C7—C12	150.6 (3)	C14—C13—C18—C17	0.0 (6)
C13—Si1—C7—C12	−87.2 (3)	Si1—C13—C18—C17	177.2 (3)
O1—Si1—C7—C8	−146.2 (3)	C24—C19—C20—C21	0.5 (7)
C1—Si1—C7—C8	−27.5 (3)	C19—C20—C21—C22	−0.7 (7)
C13—Si1—C7—C8	94.7 (3)	C20—C21—C22—C23	0.4 (6)
C12—C7—C8—C9	−1.0 (5)	C20—C21—C22—C25	−178.5 (4)
Si1—C7—C8—C9	177.2 (3)	C21—C22—C23—C24	0.2 (6)
C7—C8—C9—C10	0.2 (6)	C25—C22—C23—C24	179.0 (4)
C8—C9—C10—C11	0.9 (6)	C20—C19—C24—C23	0.1 (7)
C9—C10—C11—C12	−1.2 (6)	C22—C23—C24—C19	−0.4 (6)

Symmetry code: (i) −*x*+1, −*y*+1, −*z*+1.
